# Clinical outcome of preimplantation genetic diagnosis and screening using next generation sequencing

**DOI:** 10.1186/2047-217X-3-30

**Published:** 2014-12-04

**Authors:** Yueqiu Tan, Xuyang Yin, Shuoping Zhang, Hui Jiang, Ke Tan, Jian Li, Bo Xiong, Fei Gong, Chunlei Zhang, Xiaoyu Pan, Fang Chen, Shengpei Chen, Chun Gong, Changfu Lu, Keli Luo, Yifan Gu, Xiuqing Zhang, Wei Wang, Xun Xu, Gábor Vajta, Lars Bolund, Huanming Yang, Guangxiu Lu, Yutao Du, Ge Lin

**Affiliations:** Institute of Reproductive and Stem Cell Engineering, Central South University, Changsha, China; BGI-ShenZhen, ShenZhen, China; BGI-Health, BGI-Shenzhen, Shenzhen, China; Shenzhen Municipal Birth Defect Screening Project Lab, BGI-Shenzhen, Shenzhen, China; National Engineering and Research Center of Human Stem Cell, Changsha, China; Reproductive & Genetic Hospital of CITIC Xiangya, Changsha, China; Guangdong Enterprise Key Laboratory of Human Disease Genomics, BGI-Shenzhen, Shenzhen, China; Key Laboratory of Stem Cell and Reproductive Engineering, Ministry of Health, Changsha, China; Central Queensland University, Rockhampton, Queensland Australia; Department of Biomedicine, Aarhus University, Aarhus, Denmark; Department of Biology, University of Copenhagen, Copenhagen, Denmark; Prince Aljawhra Center of Excellence in Research of Hereditary Disorders, King Abdulaziz University, Jeddah, Saudi Arabia; James D Watson Institute of Genome Science, Hangzhou, China; School of Bioscience and Bioengineering, South China University of Technology, Guangzhou, China; State Key Laboratory of Bioelectronics, School of Biological Science and Medical Engineering, Southeast University, Nanjing, China; Section of Molecular Disease Biology, Department of Veterinary Disease Biology, Faculty of Health and Medical Sciences, University of Copenhagen, Copenhagen, Denmark

**Keywords:** Preimplantation genetic diagnosis/screening, Next generation sequencing, Blastocyst, Cryopreserved embryo transfer, Clinical outcome

## Abstract

**Background:**

Next generation sequencing (NGS) is now being used for detecting chromosomal abnormalities in blastocyst trophectoderm (TE) cells from *in vitro* fertilized embryos. However, few data are available regarding the clinical outcome, which provides vital reference for further application of the methodology. Here, we present a clinical evaluation of NGS-based preimplantation genetic diagnosis/screening (PGD/PGS) compared with single nucleotide polymorphism (SNP) array-based PGD/PGS as a control.

**Results:**

A total of 395 couples participated. They were carriers of either translocation or inversion mutations, or were patients with recurrent miscarriage and/or advanced maternal age. A total of 1,512 blastocysts were biopsied on D5 after fertilization, with 1,058 blastocysts set aside for SNP array testing and 454 blastocysts for NGS testing. In the NGS cycles group, the implantation, clinical pregnancy and miscarriage rates were 52.6% (60/114), 61.3% (49/80) and 14.3% (7/49), respectively. In the SNP array cycles group, the implantation, clinical pregnancy and miscarriage rates were 47.6% (139/292), 56.7% (115/203) and 14.8% (17/115), respectively. The outcome measures of both the NGS and SNP array cycles were the same with insignificant differences. There were 150 blastocysts that underwent both NGS and SNP array analysis, of which seven blastocysts were found with inconsistent signals. All other signals obtained from NGS analysis were confirmed to be accurate by validation with qPCR. The relative copy number of mitochondrial DNA (mtDNA) for each blastocyst that underwent NGS testing was evaluated, and a significant difference was found between the copy number of mtDNA for the euploid and the chromosomally abnormal blastocysts. So far, out of 42 ongoing pregnancies, 24 babies were born in NGS cycles; all of these babies are healthy and free of any developmental problems.

**Conclusions:**

This study provides the first evaluation of the clinical outcomes of NGS-based pre-implantation genetic diagnosis/screening, and shows the reliability of this method in a clinical and array-based laboratory setting. NGS provides an accurate approach to detect embryonic imbalanced segmental rearrangements, to avoid the potential risks of false signals from SNP array in this study.

**Electronic supplementary material:**

The online version of this article (doi:10.1186/2047-217X-3-30) contains supplementary material, which is available to authorized users.

## Background

Chromosomal abnormalities, including numerical errors and structural anomalies, are widespread in human embryos produced *in vitro*[1], and the incidence increases dramatically in embryos with advancing maternal age [2–4]. Chromosomal abnormalities are a main reason for spontaneous abortions [5, 6], and repeated implantation failures after transfer of *in vitro* produced embryos [7]. Furthermore, embryos from carriers of balanced translocations [8, 9] are at particularly high risk of chromosomal abnormalities. Previous papers have been published about the application of preimplantation genetic screening (PGS) in cases with advanced maternal age [10, 11] or recurrent pregnancy loss [12], and the application of preimplantation genetic diagnosis (PGD) in carriers of translocations [13]. Thus, PGD/PGS and the selection of chromosomally normal embryos for transfer should be an effective approach to improve live birth rate, as well as reduce spontaneous abortion and birth defects. Studies were performed using blastocyst biopsy [14] and either fluorescence *in situ* hybridization (FISH) [15], comparative genomic hybridization (CGH) [16–18] or array based methods, such as array-CGH [13, 19, 20] and single-nucleotide polymorphism array (SNP array) [21–24] for sample analysis. So far, FISH was the most common approach for sample analysis, although the obtainable information was limited: the FISH method could only detect a few chromosomes, and no detailed information could be obtained about the sub-chromosomal abnormalities. Array based techniques (aCGH and SNP array) have been used to screen all 24 human chromosomes. However, array-based methods are still relatively expensive, restricting their clinical applications.

Next generation sequencing (NGS) has now become cost-effective, and this precise and comprehensive genetic analysis tool is being increasingly used in human medicine including noninvasive prenatal diagnosis [25], and there is increasing interest for its application in PGD/PGS as well. Previous studies [26, 27] performed on trophectodermal biopsies have proven that aneuploid and unbalanced rearrangements can be detected accurately by NGS. However, no data are available to confirm the efficiency and clinical outcome of NGS-based PGD/PGS.

Here, we present the first clinical evaluation for NGS-based PGD/PGS combined with trophectoderm (TE) biopsy and cryopreserved embryo transfer (CET). We also employed SNP array as control for comparison in this study. The clinical outcomes including implantation, clinical pregnancy and miscarriage were recorded. In addition, the accuracy of NGS and SNP array were also evaluated in this study.

## Data description

A total of 395 couples were subjected to *in vitro* fertilization-preimplantation genetic diagnosis (IVF-PGD) treatment, including 129 couples with a NGS-based test and 266 couples with a SNP array-based test, for the detection of embryonic chromosomal abnormalities. The NGS test was performed using low coverage whole-genome sequencing with a HiSeq 2000 platform. The SNP array test was performed using Affymetrix Gene Chip Mapping Nsp I 262 K. The average age of the patients was 32.1 years (with an age range of 20–44 years). In the NGS cycles group, 84 patients were confirmed to have a chromosomally abnormal karyotype; 18 of which had a Robertsonian translocation, 59 had a reciprocal translocation, and 7 patients had a inversion. Another 45 couples were included because of an advanced maternal age (AMA; ≥38 years) and/or recurrent miscarriage (RM; ≥2). In the SNP array cycles group, 213 patients (of which 58 had a Robertsonian translocation, 144 had a reciprocal translocation, 11 had an inversion) and another 57 couples with AMA and/or RM were included. A total of 1,512 blastocysts were obtained, with 454 blastocysts for NGS testing and 1,058 blastocysts for SNP array analysis. In addition, 150 blastocysts from the 454 blastocysts were subjected to both NGS and SNP array tests, and blastocysts with inconsistent results would be further validated by qPCR.

## Analyses

### NGS and SNP array testing of blastocysts

Of the 454 blastocysts with NGS testing, an average of 8.2 million reads was obtained for each blastocyst, covering 5.5% ± 1.2% of the whole human genome and 98.7% ± 3.1% of the mitochondrial DNA (mtDNA). The ratio between the mean depth of the mitochondrial DNA and the genome for each blastocyst with NGS testing was calculated, which represented the relative copy number of mtDNA. It is displayed in Additional file 1: Table S3 as the Ratio (ChrM_depth/Mean_depth). The box plot of the data is shown in Additional file 2: Figure S2.

A total of 198 (43.6%) euploid and 256 (56.4%) chromosomally abnormal blastocysts were identified by NGS. Among the abnormalities, 85 blastocysts had numerical chromosome aberrations, 127 contained imbalanced structural aberrations, and 44 blastocysts were detected with both numerical and imbalanced structural aberrations. Among the diagnosed blastocysts, the median number of normal/balanced embryos per couple was 1.53 (range from 0 to 6). The group of reciprocal translocation carriers obtained 43.0% (105/244) embryos with imbalanced aberrations, most of the imbalances were translocation related. Normal/balanced embryos constituted 54.6% (65/119) of the cohort in couples with AMA and/or RM (Table 1).

**Table 1:** **NGS testing of the blastocysts**

Clinical data	Couples				Total
	Robertsonian translocation	Reciprocal translocation	Inversion	AMA and/or RM	
No. of couples treated	18	59	7	45	129
Maternal age (years)	33.9	31.0	33.3	36.9	33.6
No. of blastocysts biopsied	58	244	33	119	454
Euploid	31 (53.5%)	84 (34.4%)	18 (54.5%)	65 (54.6%)	198 (43.6%)
Numerical aberrations	18 (31.0%)	26 (10.7%)	8 (24.3%)	33 (27.7%)	85 (18.7%)
Imbalanced aberrations	6 (10.3%)	105 (43.0%)	6 (18.2%)	10 (8.4%)	127 (28.0%)
Numerical + imbalanced	3 (5.2%)	29 (11.9%)	1 (3.0%)	11 (9.3%)	44 (9.7%)

NGS: next generation sequencing; AMA: advanced maternal age; RM: recurrent miscarriage.

Of the 1,058 blastocysts with SNP array analysis, 468 (44.2%) euploid and 590 (55.8%) chromosomally abnormal blastocysts were identified, 189 blastocysts had numerical chromosome aberrations, 298 contained imbalanced structural aberrations and 103 blastocysts were detected with both numerical and imbalanced structural aberrations. The median number of normal/balanced embryos per couple was 1.76 (range from 0 to 8). The group of reciprocal translocation carriers obtained 40.8% (247/606) embryos with imbalanced aberrations. Normal/balanced embryos constituted 60.2% (106/176) of the cohort in couples with AMA and/or RM (Table 2).

**Table 2:** **SNP array testing of the blastocysts**

Clinical data	Couples				Total
	Robertsonian translocation	Reciprocal translocation	Inversion	AMA and/or RM	
No. of couples treated	58	144	11	53	266
Maternal age (years)	30.6	30.4	31.8	35.1	31.4
No. of blastocysts biopsied	229	606	47	176	1,058
Euploid (%)	120 (52.4%)	216 (35.6%)	26 (55.3%)	106 (60.2%)	468 (44.2%)
Numerical aberrations	72 (31.4%)	67 (11.1%)	8 (17.0%)	42 (23.9%)	189 (17.9%)
Imbalanced aberrations	22 (9.6%)	247 (40.8%)	8 (17.0%)	21 (11.9%)	298 (28.2%)
Numerical + imbalanced	15 (6.6%)	76 (12.5%)	5 (10.7%)	7 (4.0%)	103 (9.7%)

SNP: single nucleotide polymorphism; AMA: advanced maternal age; RM: recurrent miscarriage.

### Clinical outcome

Among the 129 couples in the NGS cycles group, 33 couples had no euploid embryos suitable for transfer; 75 couples underwent embryo transfer and the remaining 21 couples are currently still waiting for transfer. In the SNP array cycles group, 177 couples underwent embryo transfer, 66 couples had no suitable embryos for transfer, and 23 couples are currently still waiting.

Of the 666 normal/balanced blastocysts, 421 blastocysts were warmed after vitrification, 406 survived (96.4% of survival rate) and were transferred in 283 cycles. The numbers of blastocysts transferred per cycle were 1.425 (114/80) and 1.438 (292/203) for NGS and SNP array, respectively. The proportion of transferred embryos that successfully implanted was evaluated by ultrasound 6–7 weeks after embryo transfer, indicating that 60 and 139 embryos resulted in a fetal sac, giving implantation rates of 52.6% (60/114) and 47.6% (139/292) for NGS and SNP array, respectively. Prenatal diagnosis with karyotyping of amniocentesis fluid samples did not find any fetus with chromosomal abnormalities. A total of 164 pregnancies were detected, with 129 singletons and 35 twins. The clinical pregnancy rate per transfer cycle was 61.3% (49/80) and 56.7% (115/203) for NGS and SNP array, respectively (Table 3). A total of 24 miscarriages were detected, giving rates of 14.3% (7/49) and 14.8% (17/115) in NGS and SNP array cycles, respectively. In NGS cycles group, four miscarriages were spontaneous abortions occurred in early pregnancy, and the other three were artificial abortions with two caused by embryo diapause and another one caused by extrauterine (cervical) implantation. In the SNP array cycles group, 12 spontaneous abortions in early pregnancy and five artificial abortions occurred. Testing of the miscarried tissue by comparative genomic hybridization (CGH) revealed that the miscarried embryos in both NGS and SNP array cycles were chromosomally normal. The ongoing pregnancy rates were 52.5% (42/80) and 48.3% (98/203) in NGS and SNP array cycles, respectively. Out of these pregnancies, 24 babies were delivered in 20 NGS cycles; so far, all the babies are healthy and chromosomally normal according to karyotype analysis. In the SNP array cycles group the outcome of all pregnancies went to full term and 75 healthy babies were delivered (Table 3).

**Table 3:** **Clinical outcomes for the PGD/PGS cycles**

Clinical measures	All couples	Couples with NGS testing	Couples with SNP array testing	p-value
No. of embryos transferred	406	114	292	
No. of couples participating	395	129	266	
No. of couples transferred	252	75	177	
Transfer cycles	283	80	203	
Clinical pregnancy/Clinical pregnancy rate per ET	57.95% (164/283)	61.25% (49/80)	56.65% (115/203)	0.480
Ongoing pregnancy/Ongoing pregnancy rate	49.47% (140/283)	52.50% (42/80)	48.28% (98/203)	0.522
Miscarriage/Miscarriage rate	14.63% (24/164)	14.29% (7/49)	14.78% (17/115)	0.934
Implantation/Implantation rate	49.01% (199/406)	52.63% (60/114)	47.60% (139/292)	0.362
No. of healthy babies/delivered babies	99/99	24/24	75/75	

PGD: preimplantation genetic diagnosis; PGS: preimplantation genetic screening; NGS: next generation sequencing; SNP: single nucleotide polymorphism; ET: embryo transfer.

### Statistical analysis

The outcome measures of NGS and SNP array cycles were used for Pearson’s chi-squared test. The p-value of clinical pregnancy, ongoing pregnancy, miscarriage and implantation rates between NGS and SNP array groups were 0.480, 0.522, 0.934 and 0.362, respectively (Table 3). All of these p-values far outweighed 0.05 that does not reach a specified significance level. It can be concluded that the four outcome measures between NGS and SNP array cycles were the same with insignificant difference.

The Ratio (ChrM_depth/Mean_depth) of the euploid and the chromosomally abnormal blastocysts with NGS testing in Additional file 1: Table S3 was used for Mann–Whitney test. The p-value was 0.017, which was less than 0.05. It can be concluded that the copy number of mtDNA for the chromosomally abnormal blastocysts were significantly higher than the cohort for the euploid blastocysts by NGS testing.

### Cases with both NGS and SNP array analysis

Among the NGS-tested 460 blastocysts, 150 blastocysts were selected randomly for Affymetrix SNP array analysis as well, including 68 normal/balanced blastocysts and 82 blastocysts with chromosomal abnormalities. All of the normal/balanced blastocysts in the NGS test group were confirmed by SNP array. Among the chromosomally abnormal blastocysts, 7 blastocysts gave inconsistent results, which were further validated by qPCR. The detailed qPCR data for the 7 blastocysts can be seen in Additional file 1: Table S1. The qPCR data proved the accuracy of NGS since all the qPCR signals were in accordance with NGS (Table 4; Additional file 1: Table S1). There were 4 embryos suspected to be with false-negative signals and 2 embryos suspected to be with false-positive signals in the SNP array results, which were all detected accurately by NGS. A case with gain of Y chromosome was detected by NGS, but omitted by array for the lack of a Y chromosome marker in the SNP array.

**Table 4:** **Cases with inconsistent results between NGS and SNP array, and validation by qPCR**

Embryo	Embryo grade	Indications of PGD/PGS	NGS results	SNP-array results	qPCR validation
P05-1	5BB	46,XX,t(1;2)(q23;q31)	seq 1q25.3 → qter(181509440–246832193)×3	No aberration of 1	1(q25.3-qter) gain
			seq 2q31.3 → qter(182458386–242690112)×1	arr 2q32 → qter(180777009–242650581) ×1	
P06-1	5BB	AMA, RM	seq 2q22.1 → qter(137671536–238692973)×3	arr 2q23.1 → q36.1(149105998–223098449)×3	
			seq 5q12.3 → q35.3(65909293–180786350)×1	arr 5q23.3 → qter(131821983–180629495)×1	
			seq 8q11.23 → q23.1(53409090–109384199)×3	arr 8q12 → q23(56748900–112484426)×3	
			No aberration of 19	arr 19q13.11 → qter(38709901–63731511)×1	19q13 normal
			seq 20q13.12 → qter(45325082–60016946)×1	arr 20q11.21 → qter(30108048–62376958)×1	
P06-4	5BC	AMA, RM	seq 2pter → p22.3(1–34106490)×3	arr 2pter → p23(24048–2928815)×3	
			seq 12pter → p12.1(16037–25938212)×1	arr 12pter → p12(50446–19567738)×1	
			seq 14q11.2 → q23.3(19373466–64143228)×1	No aberration of 14	14(q11.2-q23.3) loss
			seq 16pter → qter(1606029–88555348)×3	arr 16pter → q13(31010–48646787)×3	
P07-3	3BC	46,XX,inv(6)(p21q25)	No aberration of 2	arr 2q10 → q13(94914686–110337634)×3	2(q10-q13) normal
			seq 19pter → qter(12244–63686007)×3	arr 19pter → qter(1–63686007)×3	
P21-1	5BC	46,XY,t(8;18)(p11;q21)	seq 8pter → p12(384809–31731414)×1	arr 8pter → p12(180568–33542048)×1	
			seq 18q22.1 → qter(62606544–75622883)×3	arr 18q22.3 → qter(6906594–76115554)×3	
			seq Ypter → qter(2784907–27141653)×2	No aberration of Y	Y chromosome gain
P23-5	3BC	46,XX,t(1;8)(q32;q22)	seq 4pter → p12(4–48354873)×3	No aberration of 4	4p gain
			seq 8q21.3 → q24.22(92180303–135064173)×1	arr 8q21.1-qter(113497800–146263569)×1	
			seq 12pter → qter(251302–132143742)×1	arr 12pter → qter(50446–132287718)×1	
			seq 15pter → qter(776958–99963983)×3	arr 15pter → qter(18427103–100192115)×3	
			seq 17pter → qter(444930–77158450)×1	arr 17pter → qter(18901–78599918)×1	
P24-1	5BB	46,X,inv(X)(p11q22)	seq 3pter → qter(1–199501827)×3	arr 3pter → qter(1–199501827)×3	
			seq 4pter → p15.1(3–35468007)×3	No aberration of 4	4(pter-p15.1) gain
			seq 16pter → qter(1749–88827254)×3	arr 16pter → qter(1–88827254)×3	

PGD: preimplantation genetic diagnosis; PGS: preimplantation genetic screening; NGS: next generation sequencing; SNP: single nucleotide polymorphism; qPCR: quantitative polymerase chain reaction.

## Discussion

In this study, we provide the first evaluation of clinical outcome of NGS-based PGD/PGS. According to the NGS results of 454 blastocysts, we transferred 114 normal/balanced embryos into the uterus, with implantation, clinical pregnancy and ongoing pregnancy rates of 52.6% (60/114), 61.3% (49/80) and 52.5% (42/80), respectively. Twenty-four babies were born, so far, all of them healthy, indicating the accuracy of NGS as well as the safety of blastocyst biopsy and vitrification. It illustrates that NGS-PGD is applicable for the genetically high-risk populations, such as carriers of Robertsonian translocations and reciprocal translocations. The feasibility of NGS-PGS application for the patients with AMA or RM was evaluated as well, though the benefits of aneuploidy screening for this population remain hypothetical [28, 29].

Array based methods (including aCGH and SNP arrays) provide an approach to screen all the 24 human chromosomes simultaneously, and are well established when applied to PGD/PGS – SNP array is widely used in clinics. Furthermore, an initial study [24] indicated that the pregnancy rate for translocation carriers can be increased significantly by SNP array-PGD compared with FISH-PGD.

NGS is a rapidly developing method for whole genome testing. Our previous study [26] indicated that aneuploidy and imbalanced rearrangements can be detected accurately by NGS in TE cells at a much lower depth (0.07X) of sequencing. There were 150 blastocysts with both NGS and SNP array testing in this study, 7 blastocysts were detected with inconsistent results (Table 4), and all the NGS results were confirmed to be accurate by qPCR validation. NGS provides high accuracy in the detection of some imbalanced segmental rearrangements due to the ability for NGS to correct the bias from WGA [27].

The outcome measures of NGS cycles were the same as SNP array, that the differences of the outcome measures were insignificant between these two methods. The numbers of normal/balanced embryos per couple and transferred blastocysts per cycle for the two methods were also insignificantly different. From the 150 blastocysts with both NGS and SNP array testing, we can find that the same normal/balanced embryos were detected by NGS and SNP array. Thus, the efficiency and accuracy for embryo selection would be similar between NGS and SNP array cycles, as well as the clinical outcome measures.

There were 6 embryos with signals to be inconsistent with NGS and qPCR results in SNP array data. These signals may due to the fact that only a maternal or a paternal allele is present in SNP array results and so forth, which can easily lead to false-negatives and false-positives. A case of a Y chromosome gain was detected by NGS, but omitted by array for the lack of a Y chromosome marker in SNP array. False-negatives in PGD/PGS clinical practice may lead to a higher rate of spontaneous abortion and/or birth defect [30]. False-positives in the clinical application may cause a lower rate of clinical pregnancy or implantation [31, 32]. The differences of outcome measures between NGS and SNP array cycles were insignificant, which may be due to the fact that most inexact signals of SNP array did not impact the efficiency of embryo selection in this study. Nevertheless, NGS was able to detect some segmental imbalances more precisely. So that the potential risks from false-negative and false-positive results can be avoided by the NGS test.

NGS is with a bright prospect. A case report described the use of NGS for PGD recently [33]. Several comments for the application of NGS/MPS in PGD/PGS were published [34, 35]. The cost and time of sequencing is already competitive with array tests, and the estimated reagent cost of sequencing for the detection of chromosomal abnormalities is currently less than $100. Furthermore, the cost-effectiveness of sequencing is rapidly increasing and so is the accuracy due to continuous technical improvements, including some bench-top sequencing platforms [36]. NGS is able to achieve high coverage of mtDNA in this study. The relative copy number of mtDNA for the blastocysts with NGS testing was evaluated and a significant difference was found between the copy number of mtDNA for the euploid blastocysts and the chromosomally abnormal blastocysts. It indicated the potential of embryonic mitochondria analysis by NGS to evaluate the potential for embryonic development [37]. Single-cell level-based methods using NGS is developing rapidly, such as single-cell exome sequencing [38, 39], single-cell genome analysis [40], single-cell structural variation analysis [41], and single-cell RNA sequencing [42]. These methods hold promise for the further application of NGS in embryology and reproductive medicine.

In conclusion, NGS-based PGD/PGS combined with blastocyst biopsy and vitrification can be efficiently applied in clinical practice. In this study, the clinical outcome measures of the NGS cycles were the same as SNP array, and NGS was able to detect some segmental imbalances that may be omitted by SNP array, which may avoid the potential risks of false signals. NGS-based PGD/PGS should be suitable for more extensive applications and might even provide services for the populations with genetic risks.

## Methods

### Patients

Patients with various conditions including Robertsonian translocation, reciprocal translocation, inversion, advanced maternal age (AMA) and/or with recurrent miscarriage (RM) were recruited in the PGD/PGS program at Reproductive & Genetic Hospital of CITIC Xiangya, China from August 2011 to the current date. SNP array-based PGD/PGS cycles were performed earlier than NGS-based PGD/PGS cycles in this study. The karyotype of these patients was determined by G-banding by karyotyping peripheral blood. Blastocyst samples from all couples were obtained with Institutional Review Board (IRB) approval from BGI-Shenzhen, China and Reproductive & Genetic Hospital of CITIC Xiangya, China with reference number of LL-SC-RG-2012-001 for the IRB approval, and all couples signed a written informed consent form (Additional file 3).

### Controlled ovarian hyperstimulation and fertilization

Pituitary desensitization was performed using either a long luteal Gonadotropin-releasing hormone (GnRH) agonist protocol or an antagonist protocol [43, 44] based on patient situations. Oocyte retrieval (OR) was performed 34–36 hours after human chorionic gonadotropin (hCG) injection under general anaesthesia. All eggs were fertilized by intracytoplasmic sperm injection (ICSI) 4–6 hours after OR, and normal fertilization was identified 16–18 hours after injection by the presence of two pronuclei and two polar bodies.

### Embryo culture and biopsy

All embryos were cultured in sequential media (G1 and G2, Vitrolife, Goteborg, Sweden) to the blastocyst stage. On D4 after fertilization, an 18 μm hole was made in the zona pellucida of all embryos. On D5 or D6, blastocysts in which trophectoderm (TE) cells had herniated out of the zona pellucida were chosen for biopsy. Approximately 3–8 TE cells were aspirated using a biopsy pipette with 30 μm internal diameter and dissected with a Zilos TK laser (Hamilton Thorne, MA, USA). Biopsied TE cells were washed in G-MOPS medium (Vitrolife, Goteborg, Sweden). They were either used directly for Whole Genome Amplification (WGA) or stored at -20°C until WGA.

### Next generation sequencing and data analysis

The biopsied TE cells were used for WGA with WGA4 Kit (Sigma Aldrich Co., St. Louis, USA) following the manufacturer’s instructions. 1-2ug of the WGA product was used for library construction according to the guidelines of TruSeq DNA Sample Prep Kits (Illumina, San Diego, USA) with a 350 bp insert size. The libraries were processed for single-end 50 bp read length sequencing using the Illumina HiSeq2000 platform.

The raw sequencing data were processed and quality controlled. The initial 20 bp sequence of each read was omitted, and the rest of the read sequence was aligned to a Human reference genome (Hg18; NCBI Build36) using the Short Oligonucleotide Analysis Package 2 (SOAP2) [45]. After alignment, we used an algorithm developed in-house for chromosomal abnormality analysis. The procedure involved GC correction for WGA-induced bias removal, binary segmentation for locating copy number variations (CNV) breakpoints, and dynamic threshold determination for filtering of final signals [27]. The CNVs larger than 1 megabase (>1 M) would be detected theoretically. All the results were visualized by digital karyotyping for better presentation [46].

The relative copy number of mtDNA for each blastocyst with NGS testing was calculated as the ratio between the mean depth of the mitochondrial DNA and the genome, which was displayed in Additional file 1: Table S3 as the Ratio (ChrM_depth/Mean_depth) and in Additional file 2: Figure S2 as the box plot figure. The Ratio (ChrM_depth/Mean_depth) of the euploid and the chromosomally abnormal blastocysts with NGS testing were used for Mann–Whitney test.

### SNP array testing

WGA products were also processed for the SNP array analysis according to previous reports [21, 24]. The amplified DNA of individual embryo was hybridized to the Gene Chip Mapping Nsp I 262 K microarray (Affymetrix Inc.). Approximately 260,000 SNP signals for each sample were used for copy number analysis by Gene Chip Genotyping Analysis Software (GTYPE; Affymetrix Inc) using a smoothing size of 16 Mb to eliminate background signals. As the SNP array is unable to detect the Y chromosome, Y chromosome-specific PCR was performed for sex determination and detection of Y chromosomal abnormalities. The SNP array method had been validated before clinical application and small numbers of cells from hESC lines were used, which was described in the report [24].

### qPCR validation

qPCR was used to validate the results of NGS and SNP array. It was performed according to previous protocols [47]. The WGA products of 2 YH (46, XY) single cells were used as normal control and 3 pairs of primers in chromosome 9, 12, 22 respectively were selected as internal control (Additional file 1: Table S1). Two positive samples from 2 single cells of cell lines with known karyotype as 47, XY, +21 and 45,XX,-14 were validated before clinical application (Additional file 1: Table S2). The qPCR was performed with known results of NGS and SNP arrays rather than blinded.

### Blastocysts vitrification and warming

Blastocysts were vitrified after the biopsy using Kitazato vitrification solution (Kitazato Biopharma Co. Ltd. Shizuoka. Japan) and closed High Security Vitrification straws (Cryo Bio System, France). The vitrification and warming procedure was carried out according to the protocol recommended in the Kitazato vitrification kit. Each blastocyst was stored in an individual straw. After warming and dilution, blastocysts were cultured in blastocyst medium for 1–2 hours. We selected the chromosomally normal/balanced blastocysts for warming, and the surviving re-expanded blastocysts with high morphology grade were selected for transfer.

### Luteal support and blastocyst transfer

Luteal support was applied in cryopreserved embryo transfer (CET) cycles. Warmed blastocysts were transferred either 5 days after ovulation of a natural cycle or 5 days after the initiation by progesterone. Briefly, 6 mg Estradiol Valerate was started from Day 3 for 10–15 days, then luteal support was applied when a satisfactory endometrial development (thickness ≥8 mm) was confirmed with ultrasound. No more than two blastocysts were transferred, and single blastocyst transfer to each patient with well cryopreserved embryos was recommended.

### Clinical outcome

The main outcome measures included clinical pregnancy, implantation, miscarriage and ongoing pregnancy rates. These four outcome measures of NGS and SNP array cycles were used for Pearson’s chi-squared test (-test) to evaluate the difference between the two groups. Clinical pregnancy was confirmed when an intrauterine gestational sac with heart-beat was observed by ultrasound examination 30–40 days after embryo transfer. The amniocentesis fluid samples from fetuses or the peripheral blood samples from delivered babies were used for karyotyping to confirm the PGD/PGS results.

## Availability of supporting data

The data sets supporting the results of this article are available in the European Genome-Phenome Archive repository at accession EGAD00001001037. Further details on data access are available from the GigaScience database [48].

## Electronic supplementary material

Additional file 1: **Supplementary data of sequencing, array and qPCR tests for the 7 embryos as well as the mitochondrial DNA analysis in sequencing.** (XLSX 177 KB)

Additional file 2: **Supplementary data of chromosomal abnormalities for each embryo by sequencing and array tests as well as clinical outcome for each couple.** (DOCX 1 MB)

Additional file 3: **Informed consent for preimplantation genetic diagnosis/screening.** (DOCX 33 KB)

## Abbreviations

AMA: Advanced maternal age
CET: Cryopreserved embryo transfer
CGH: Comparative genomic hybridization
CNV: Copy number variation
FISH: Fluorescence *in situ* hybridization
GnRH: Gonadotropin-releasing hormone
hCG: Human chorionic gonadotropin
ICSI: Intracytoplasmic sperm injection
IRB: Institutional Review Board
IVF: *In vitro* fertilization
mtDNA: Mitochondrial DNA
NGS: Next generation sequencing
OR: Oocyte retrieval
PGD: Preimplantation genetic diagnosis
PGS: Preimplantation genetic screening
qPCR: Quantitative polymerase chain reaction
RM: Recurrent miscarriage
SNP: Single nucleotide polymorphism
TE: Trophectoderm
WGA: Whole genome amplification.
