# Supplementary material for: Clinical outcome of preimplantation genetic diagnosis and screening using next generation sequencing
Source: Gigascience. 2014 Dec 4;3:30. doi: 10.1186/2047-217X-3-30 (PMC4326468; doi:10.1186/2047-217X-3-30)

Supplementary Figure 1. The data of sequencing and SNP array for each of the 7 discordant embryos

P05-1:

Digital karyotyping of sequencing


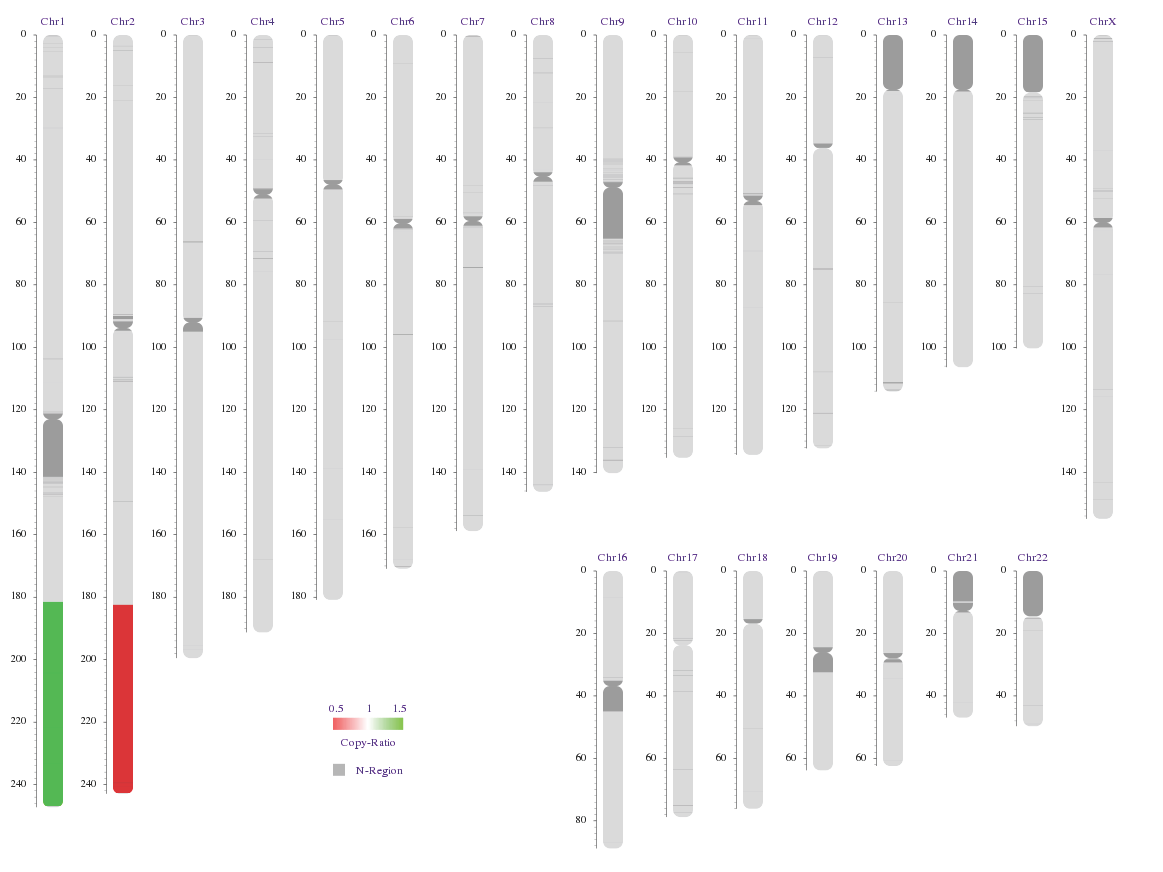


SNP array


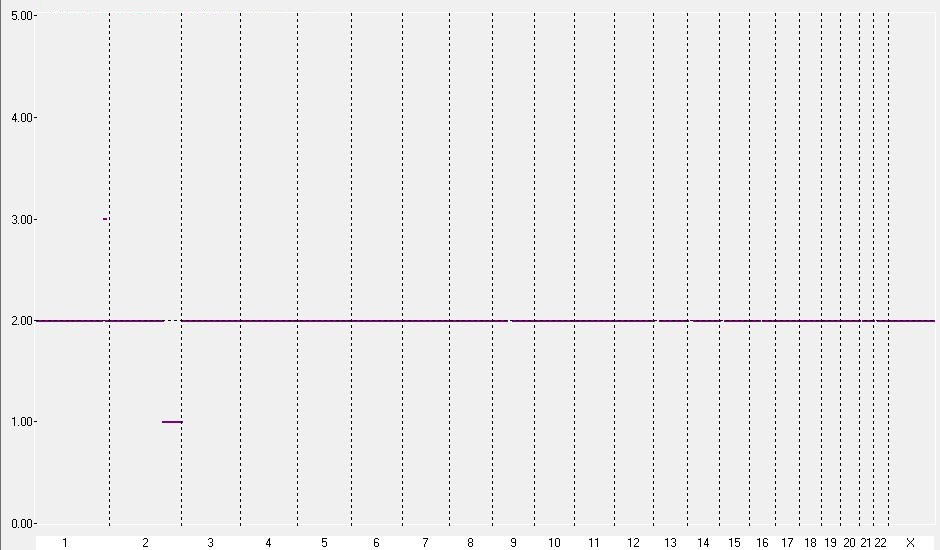


P06-1:

Digital karyotyping of sequencing


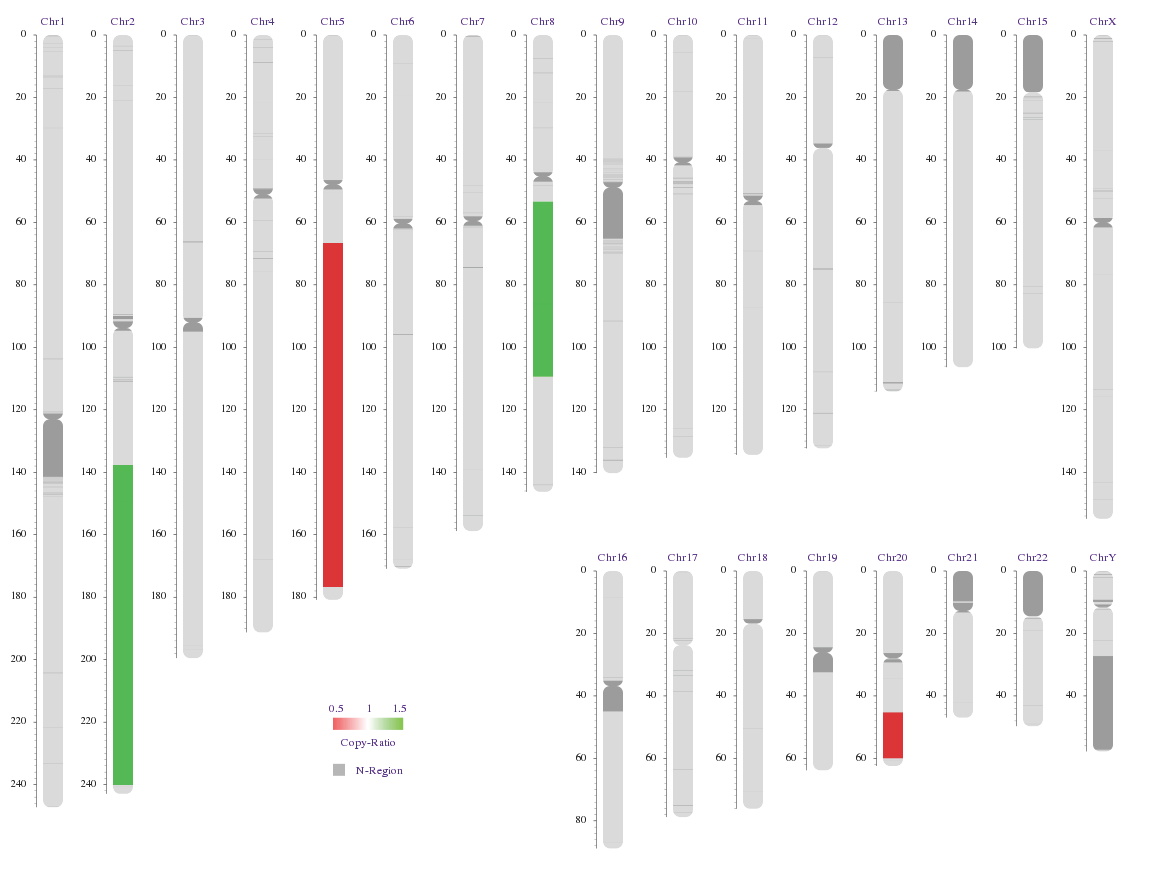


SNP array


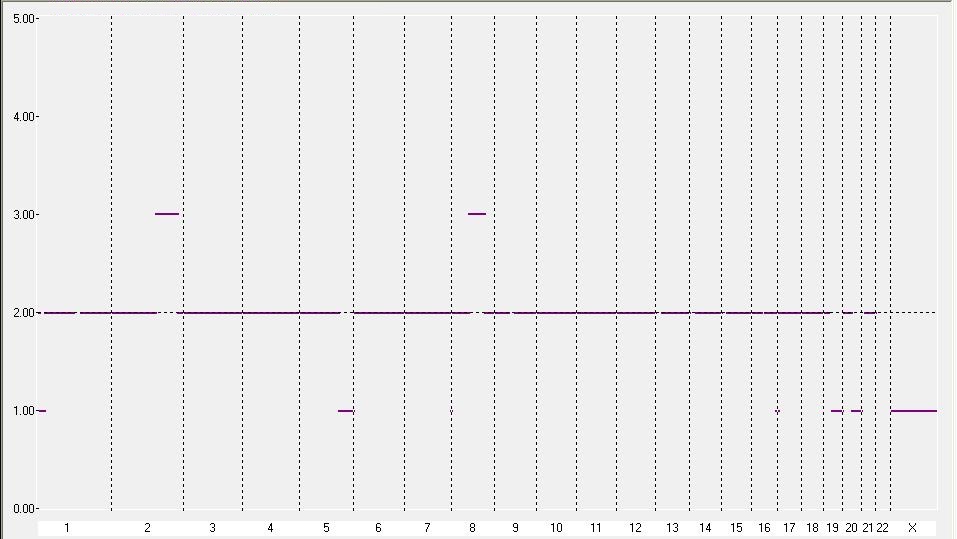


P06-4:

Digital karyotyping of sequencing


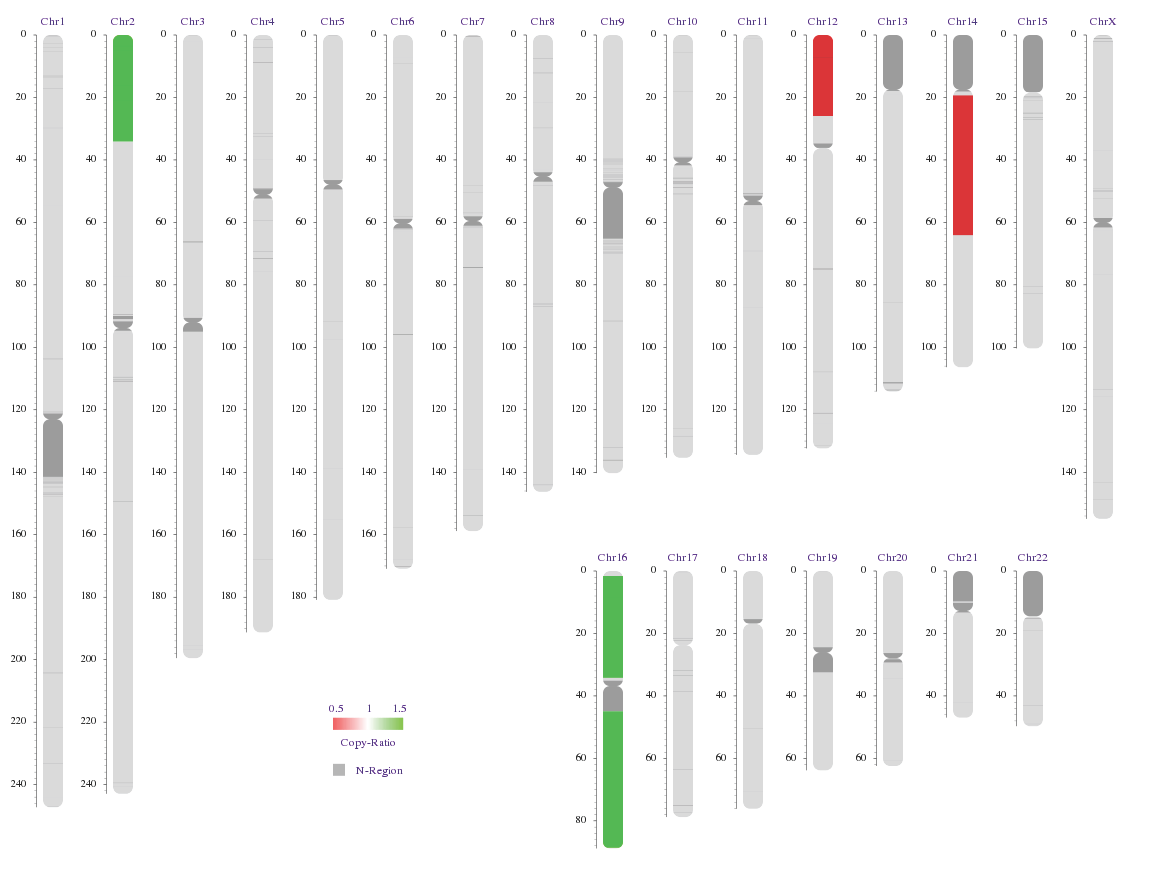


SNP array


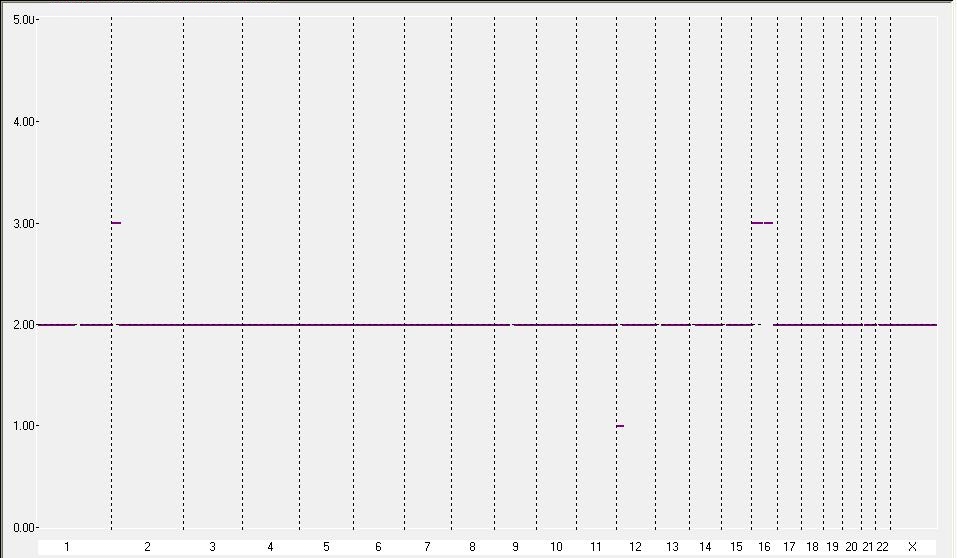


P07-3:

Digital karyotyping of sequencing


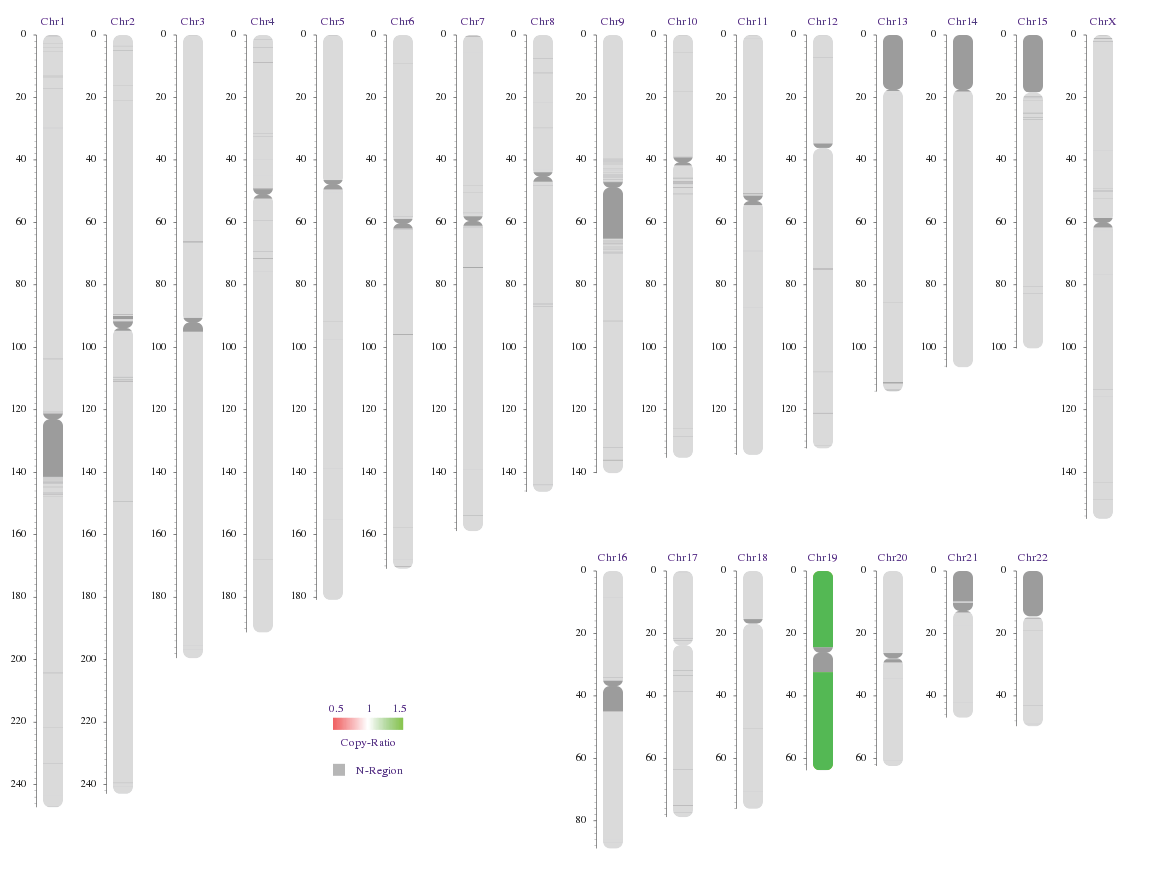


SNP array


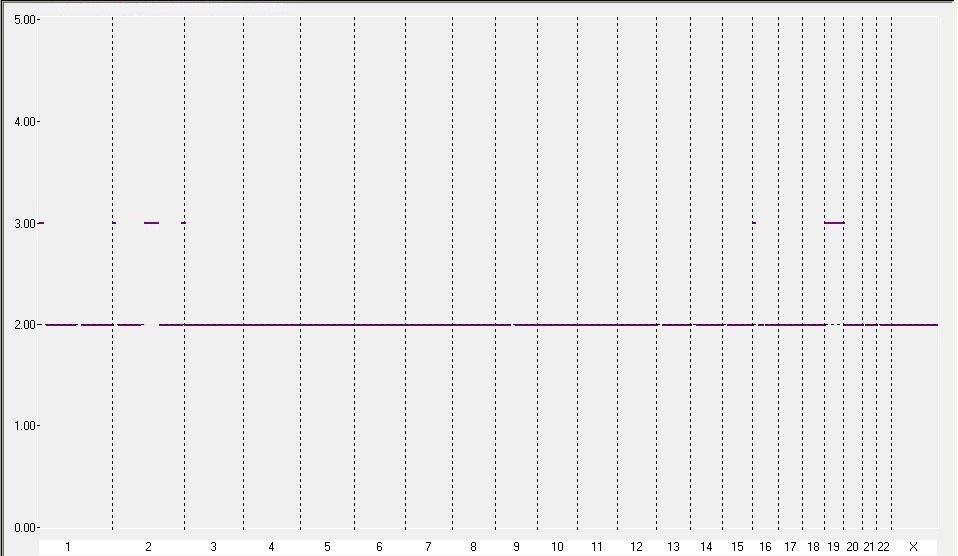


P21-1:

Digital karyotyping of sequencing


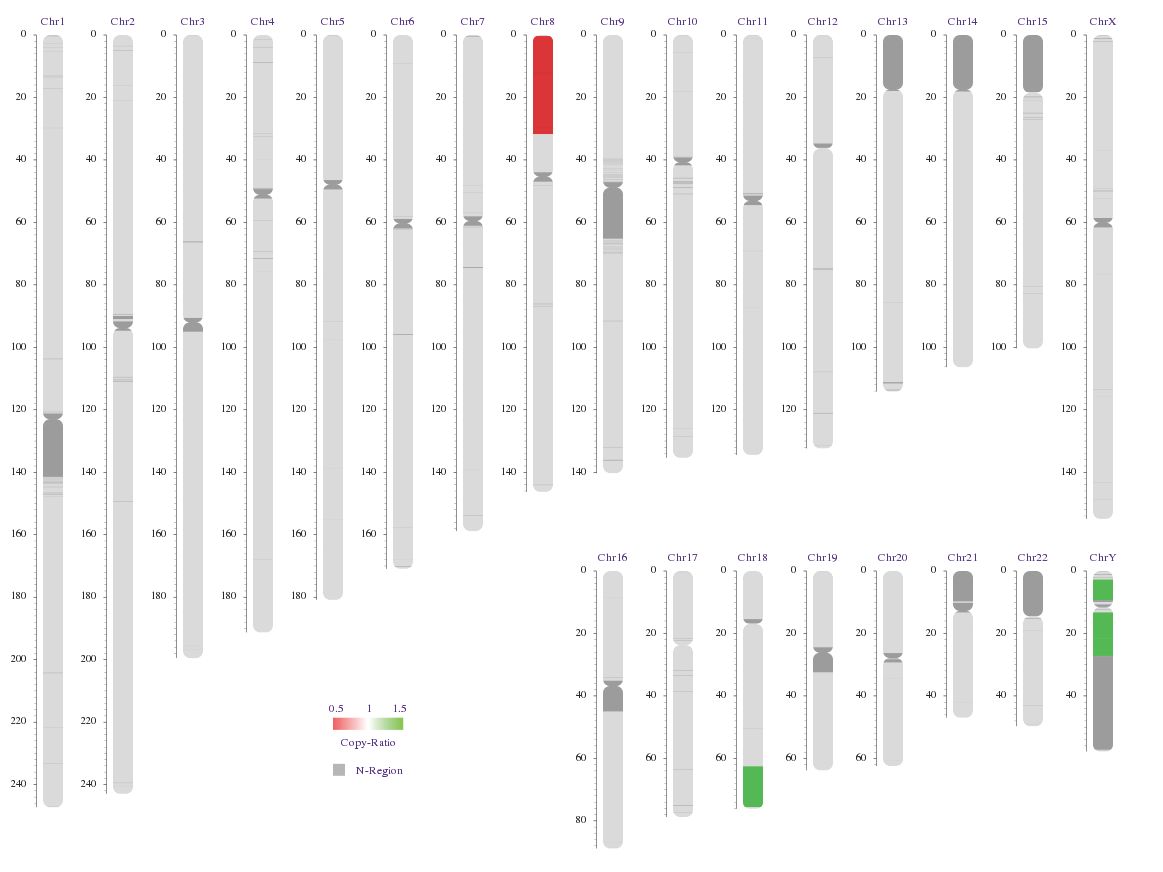


SNP array


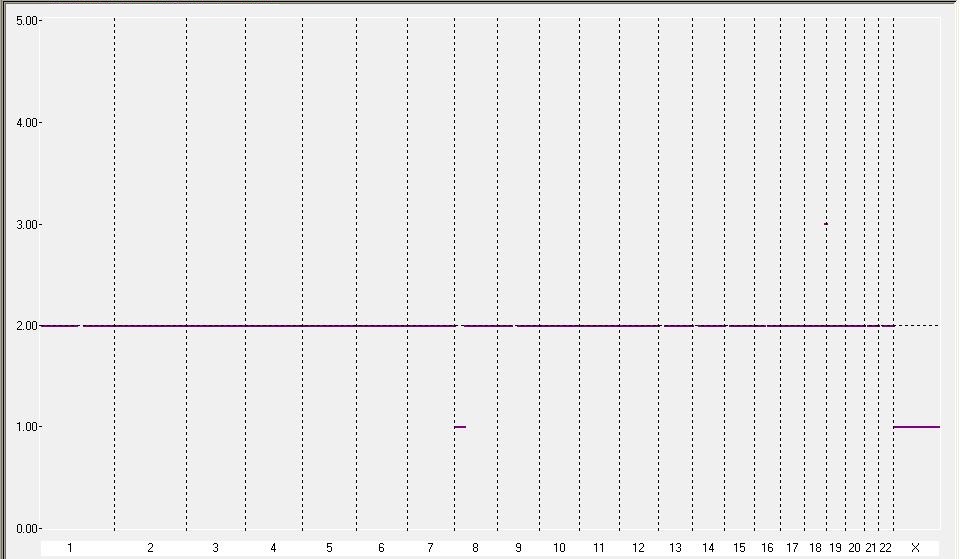


P23-5:

Digital karyotyping of sequencing


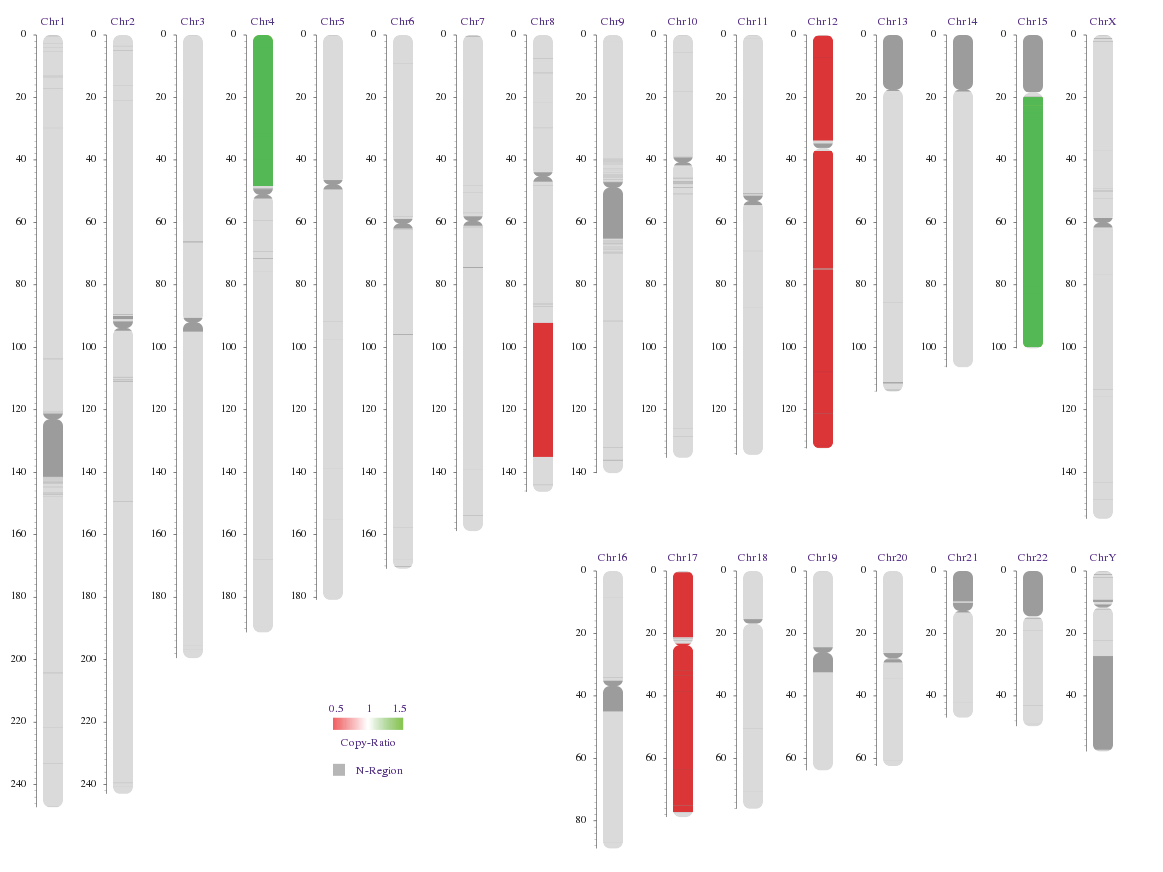


SNP array


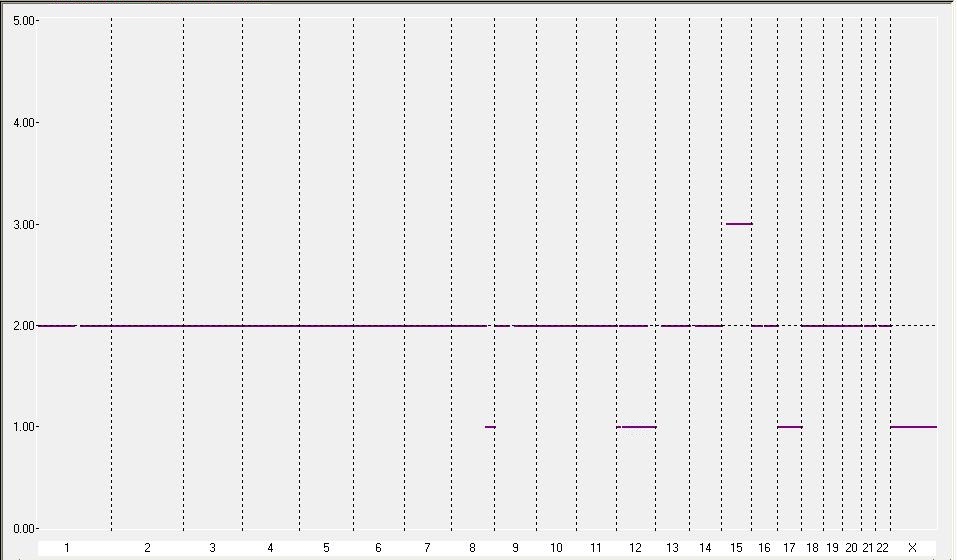


P24-1:

Digital karyotyping of sequencing


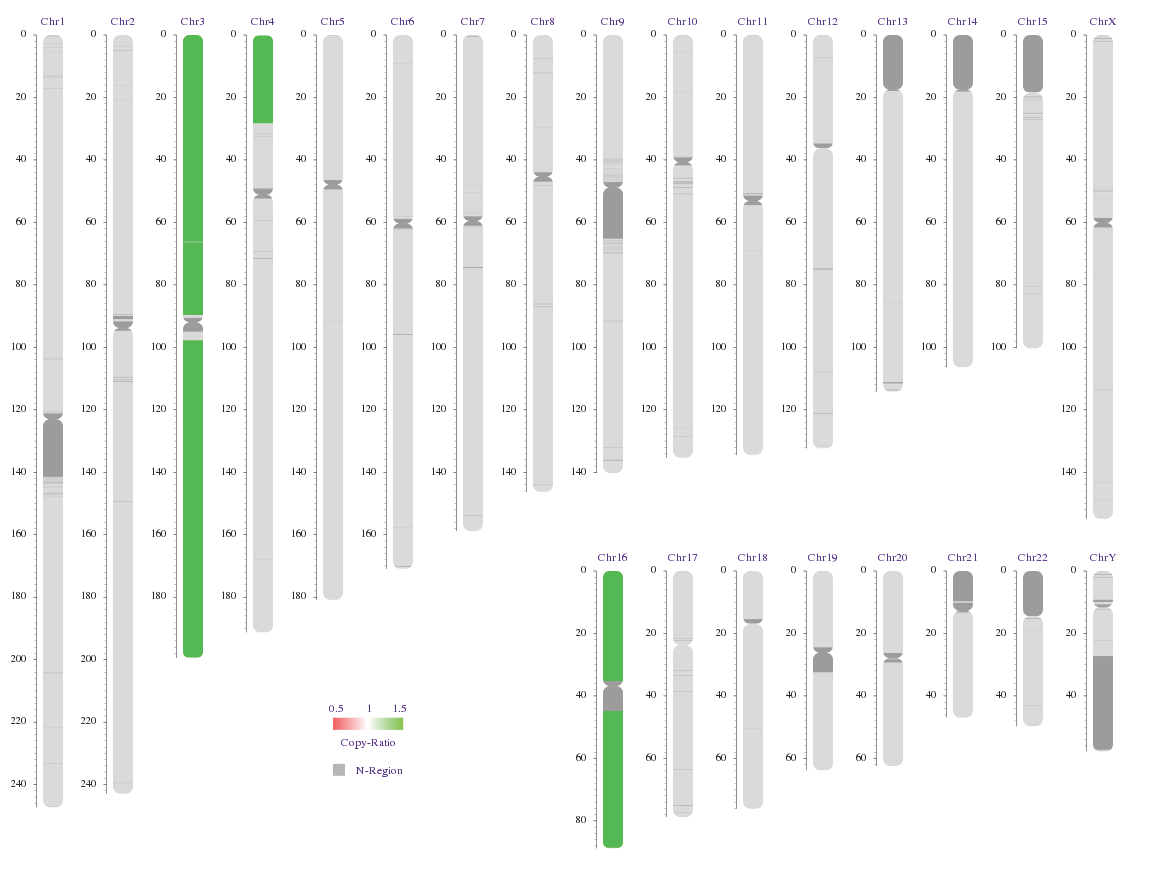


SNP array


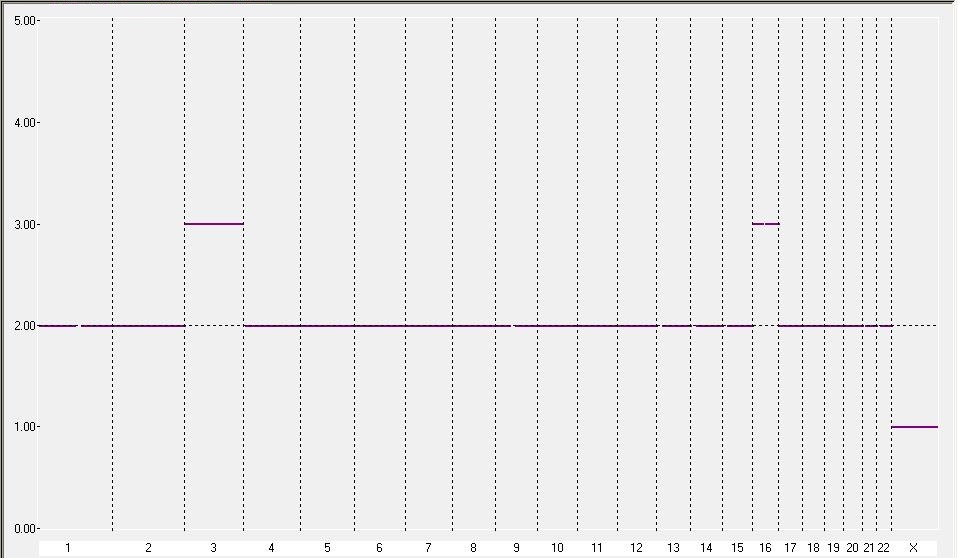


Supplementary Table 1. Data of qPCR validation for the 7 discordant embryos

| Embryo | Chromosome position | Primers | qPCR fold change (2^-ΔΔCT) | qPCR results |
| --- | --- | --- | --- | --- |
| P05-1 | 1q25.3→qter | Chr1-1F:CCGGCCGAGGTCTGGGAGAT | 1.6324 | 1(q25.3-qter) gain |
|  |  | Chr1-1R:AGAGTTCGGGACGGGAGCGG |  |  |
|  |  | Chr1-2F:TGTGGCGCTGCCCATCGTAG | 2.2292 |  |
|  |  | Chr1-2R:AGGCAGGAGTTCTGCGGGGG |  |  |
|  |  | Chr1-3F:CGGCCGAGGTCTGGGAGATG | 1.3912 |  |
|  |  | Chr1-3R:AGAGTTCGGGACGGGAGCGG |  |  |
| P06-1 | 19q13.11→qter | Chr19-1F:CTGGGAAGGGGGAGGCATGG | 0.8521 | 19q13 normal |
|  |  | Chr19-1R:GGGGAGAGCAGAGCAGGCGA |  |  |
|  |  | Chr19-2F:CCAGCTGCGGCCTTGAGGAA | 1.0070 |  |
|  |  | Chr19-2R:TGGCTGCTCGGGGCTCTGTT |  |  |
|  |  | Chr19-3F:AACAGAGCCCCGAGCAGCCA | failed |  |
|  |  | Chr19-3R:TCCCAGGCCCTGTCCTCCCT |  |  |
| P06-4 | 14q11.2→q23.3 | Chr14-1F:CTGCCAGTGCCAACCCAGCA | 0.6012 | 14(q11.2-q23.3) loss |
|  |  | Chr14-1R:ATGTCCCCGCCGTACCACCA |  |  |
|  |  | Chr14-2F:TGGTGGTACGGCGGGGACAT | failed |  |
|  |  | Chr14-2R:GCAGCTGCCAGTGCCAACCC |  |  |
|  |  | Chr14-3F:GCAGTCGCACCTCCTCGGCT | 0.3508 |  |
|  |  | Chr14-3R:GGCAGCGCCTTCGCATCTGT |  |  |
| P07-3 | 2q10→q13 | Chr2-1F:CCCTGCCCAGTGGCTTCTCG | 1.2145 | 2(q10-q13) normal |
|  |  | Chr2-1R:CTCAGCCCACCCCGTCCCTT |  |  |
|  |  | Chr2-2F:CACCGCCGTCACCCTCATCA | 0.9898 |  |
|  |  | Chr2-2R:GCCACGCCCTCCAGGACAAA |  |  |
|  |  | Chr2-3F:CGCTCGGCCATCAACCTGCT | 0.9954 |  |
|  |  | Chr2-3R:GTGACGGCGGTGAAGGGCAT |  |  |
| P21-1 | Y chromosome | ChrY-1F:GTGCAACGGATGCAGACAGTG | 1.8342 | Y chromosome gain |
|  |  | ChrY-1R:AGCATGCCTGTACGACGATCC |  |  |
|  |  | ChrY-2F:GAAAGGGCTGTGGTCTGTGGA | 2.1795 |  |
|  |  | ChrY-2R:TTATGTCTCAGGTGGGCCGAG |  |  |
| P23-5 | 4pter→p12 | Chr4-1F:CACCCTGGGAGTTAGGCAGAGCA | 2.1086 | 4p gain |
|  |  | Chr4-1R:CATTTGACGTGTCGGGGGAAGG |  |  |
|  |  | Chr4-2F:CACCCTGGGAGTTAGGCAGAGCA | 1.4142 |  |
|  |  | Chr4-2R:GCAAGTCATTTGACGTGTCGGGG |  |  |
|  |  | Chr4-3F:CACCCTGGGAGTTAGGCAGAGCA | 2.0018 |  |
|  |  | Chr4-3R:TCATTTGACGTGTCGGGGGAA |  |  |
| P24-1 | 4pter→p15.1 | Chr4-1F:CACCCTGGGAGTTAGGCAGAGCA | 1.5956 | 4(pter-p15.1) gain |
|  |  | Chr4-1R:CATTTGACGTGTCGGGGGAAGG |  |  |
|  |  | Chr4-2F:CACCCTGGGAGTTAGGCAGAGCA | 2.8630 |  |
|  |  | Chr4-2R:GCAAGTCATTTGACGTGTCGGGG |  |  |
|  |  | Chr4-3F:CACCCTGGGAGTTAGGCAGAGCA | 2.9607 |  |
|  |  | Chr4-3R:TCATTTGACGTGTCGGGGGAA |  |  |
| Internal control | chromosome 9 | Chr9-1F:ATCCTCAATAGCCAGGCCCAA | 1.0000 |  |
|  |  | Chr9-1R:GCTAAATGGCTGCAGTGTGGG |  |  |
|  | chromosome 12 | Chr12-1F:TTTGGCCTTGGATTTGGGAGT |  |  |
|  |  | Chr12-1R:CGACGGCACTGGAAAATCTTG |  |  |
|  | chromosome 22 | Chr22-1F:TAGTGAATCACCTGGCAGCCC |  |  |
|  |  | Chr22-1R:CGGTCTACCACTGGAGCATGG |  |  |

Supplementary Table 2. Data of qPCR for the 2 positive samples before clinical application.

| Sample | Chromosome position | Primers | qPCR fold change (2^-ΔΔCT) | qPCR results |
| --- | --- | --- | --- | --- |
| 47,XY,+21 | Chromosome 21 | Chr21-1F:CTCTGGTGCCTTAGTCCCGGT | 1.7868 | trisomy 21 |
|  |  | Chr21-1R:TGGACTACTGCTCCGCAGGAC |  |  |
|  |  | Chr21-2F:CATGGGTTTTGCAGCCTGTTC | failed |  |
|  |  | Chr21-2R:AAAAGGAAAGTGCTGGGGCAA |  |  |
|  |  | Chr21-3F:AGCTACTGCTGGGAGGTTGGG | 1.6447 |  |
|  |  | Chr21-3R:CCTGGAGCCAGCTTCCACTTT |  |  |
| 45,XX,-14 | Chromosome 14 | Chr14-1F:TGCTTTGGCCTGAGGTTGTTC | 0.1048 | monosomy 14 |
|  |  | Chr14-1R:GGTTAAACCAGGGGGCCATTT |  |  |
|  |  | Chr14-2F:GAAAGAGCTTTGTGCGAGGCA | 0.0723 |  |
|  |  | Chr14-2R:TCCTTGAAGGGGACCAGAAGC |  |  |
|  |  | Chr14-3F:AATGGGGCAGAATGCTGTTGA | 0.1952 |  |
|  |  | Chr14-3R:CCCTGCCAAGTTCCTTGACCT |  |  |
|  | Chromosome X | ChrX-1F:ACCCGGGGATCCTCTATGTCA | 1.7985 | XX |
|  |  | ChrX-1R:GGAACGTTTCGCATGGACTTG |  |  |
|  |  | ChrX-2F:CAGAACCCCGCTCGGTTTATC | 2.2506 |  |
|  |  | ChrX-2R:GCAGGTGTGTTTGGGATGGAG |  |  |
|  |  | ChrX-3F:GAACTGAGCGGGCAGAGTTGA | 1.9563 |  |
|  |  | ChrX-3R:GCCCAGAGTGGTCACTGGGTA |  |  |
| Internal control | chromosome 9 | Chr9-1F:ATCCTCAATAGCCAGGCCCAA | 1.0000 |  |
|  |  | Chr9-1R:GCTAAATGGCTGCAGTGTGGG |  |  |
|  | chromosome 12 | Chr12-1F:TTTGGCCTTGGATTTGGGAGT |  |  |
|  |  | Chr12-1R:CGACGGCACTGGAAAATCTTG |  |  |
|  | chromosome 22 | Chr22-1F:TAGTGAATCACCTGGCAGCCC |  |  |
|  |  | Chr22-1R:CGGTCTACCACTGGAGCATGG |  |  |

The 2 positive samples were WGA products from 2 single cell of cell lines with known karyotype as 47,XY,+21 and 45,XX,-14.

Supplementary Figure 2. Box plot of the relative copy number of mitochondrial DNA for chromosomally abnormal and euploid blastocysts with NGS testing


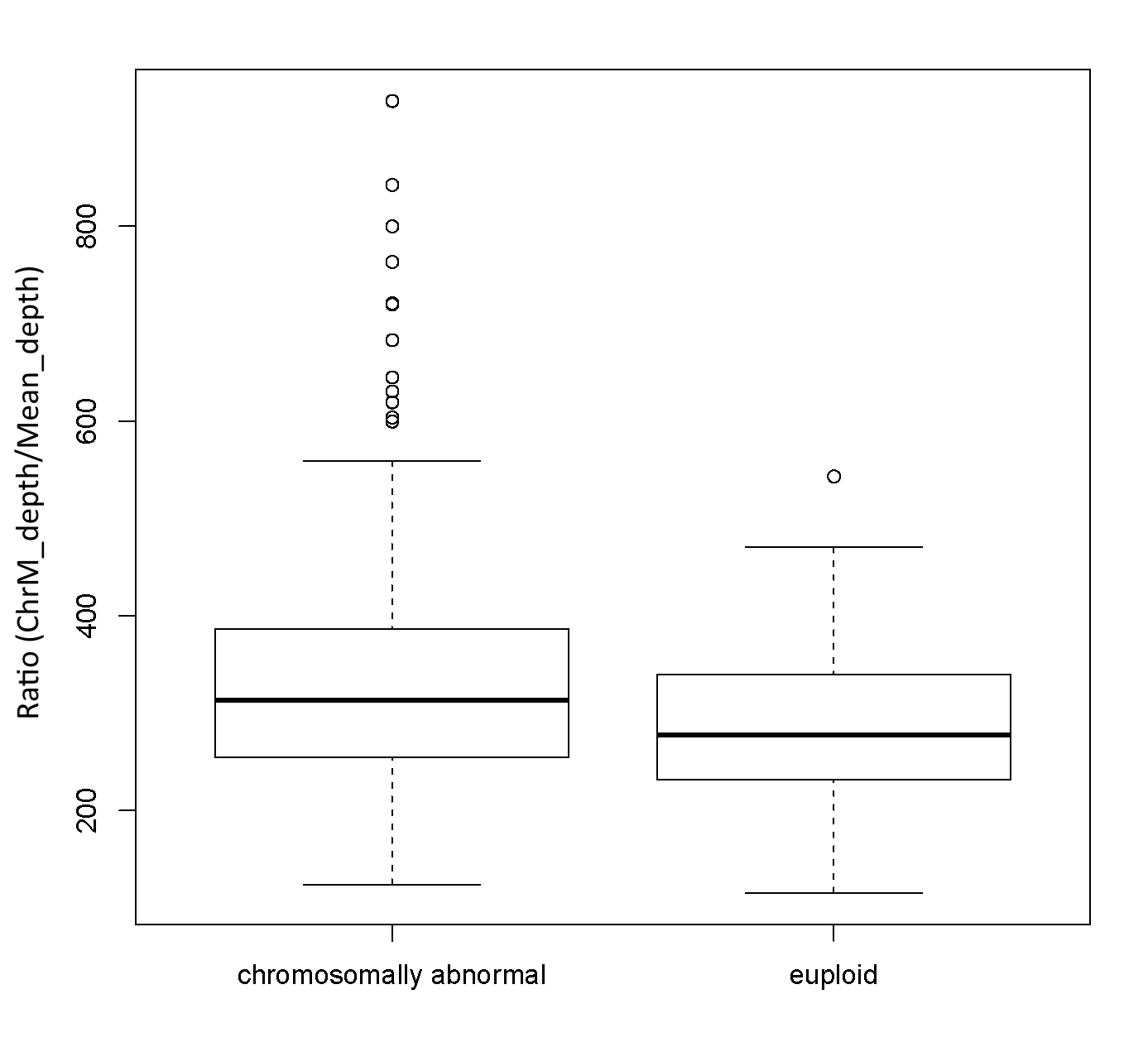

Supplement: Supplementary file 2 — Additional file 2: Supplementary data of chromosomal abnormalities for each embryo by sequencing and array tests as well as clinical outcome for each couple. (DOCX 1 MB) [file 13742_2014_61_MOESM2_ESM.docx]
