# Supplementary material for: Clinical outcome of preimplantation genetic diagnosis and screening using next generation sequencing
Source: Gigascience. 2014 Dec 4;3:30. doi: 10.1186/2047-217X-3-30 (PMC4326468; doi:10.1186/2047-217X-3-30)
Supplement: Supplementary file 3 — Additional file 3: Informed consent for preimplantation genetic diagnosis/screening. (DOCX 33 KB) [file 13742_2014_61_MOESM3_ESM.docx]

**Informed Consent for Preimplantation Genetic Diagnosis/Screening**

HC-RHC

Sample barcode

Chromosomal abnormality is the major causes of early pregnancy spontaneous abortion and birth defects. It not only brings a great mental suffering and economic pressures to patients and families, but also gives a heavy burden on society. There is no effective therapy for such diseases with Modern medicine. It can only be avoided by genetic counselling, pre-implantation genetic screening and diagnosis and intervention at the early time.

BGI clinical laboratoris can analyze embryonic chromosomal aneuploidy and large segments of deletions/duplications using embryonic cells whole genome amplification combined with genome-wide low coverage sequencing method. The detection procedure can be used for pre-implantation genetic diagnosis or screening, which helps select good embryo for implantation and improve the success rate of in vitro fertilization.

I, ________________ (printed name), hereby agree to participate in testing for Pre-implantation Genetic Diagnosis/Screening using Whole genome sequencing technology. I understand that DNA will be extracted from embryonic cells for the purpose of high-throughput sequencing and bioinformatics analysis in order to identify possible chromosome aneuploidy and large segments of deletions/duplications. I hereby give permission to collect biological samples from my embryonic cells for pre-implantation genetic diagnosis.

| The limitations, benefits, nature, aims and the risks of this test have been explained to me and I understand that: |
| --- |

1. This testing method is applicable for embryo differentiation cells samples, Due to sampling techniques and whole genome amplification restrictions, there are maybe some risk of failure.
2. In view of the current limits of medical molecular detection technology, even though the inspectors had discharged its responsibilities and conduct the experimental procedures in high standard, there could be a false-positive or false-negative results.
3. I know the test can only detect selected embryos for chromosomal aneuploidy and large chromosomal deletions and duplications testing ,I am not promised a 100% accuracy of this test
4. Due to irresistible factors (such as sending delays, sample tube rupture, experiment reagents, etc.) results in sample loss may affect the sample testing.
5. This test and involved instruments are not applied for regular clinical diagnostic purpose. Currently this test is designed for assisting clinical diagnosis or medical research purpose. The result of this test only provides references for clinician/genetic counselor/physician rather than final diagnostic conclusions.
6. All test results are treated with standard medical confidentiality, and will be reported only through a physician, genetic counselor, or other identified healthcare provider. The results are confidential to the extent allowed by law. They will only be released to other medical professionals or other parties with my written consent or as otherwise allowd by law. If an insurance provider requires test results for reimbursement purpose, the laboratory is obligated to release them.
7. Genetic testing and its outcome may involve in emotional stress. BGI Clinical Laboratories will not be reposnible for the resulting consequences.
8. There will be a fee for this genetic testing. I will be responsible for the payment of this test, even if I decide not to receive result reports.
9. The laboratory does not return the remaining samples to individuals or physicians. However, in some cases, it may be possible to perform additional studies for clinical validation or medical research purposes using the remaining sample in an anonymous way. Refusal to permit the use of my sample will not affect my test result. For such use, samples will be retained in the laboratory in accordance with the laboratory retention policy. I can withdraw my consent at any time by checking this box □, and the remaining samples shall be properly destroyed.

| Patient’s/ Guardian’s Statement |
| --- |

My signature below acknowledges my voluntary participation in this test. I am fully aware of the contents above, and agree to take full responsibility for my action. I declare that the information I provided is true, intact and up-to-date.

Patient/ Guardian Signature:______________ Date:_____________

Patient/ Guardian Signature:______________ Date:_____________

| Physician’s/Genetic Counselor’s Statement |
| --- |

I have explained genetic testing (including the risk, benefits, and alternatives) to this individual and/or his/her legal guardian. I have addressed the limitations outlined above, and I have answered this person’s questions. I have obtained consent from the patient or the legal guardian for this testing.

Signature: Date:_____________
